# Threshold-Responsive Colorimetric Sensing System for the Continuous Monitoring of Gases

**DOI:** 10.3390/s23073496

**Published:** 2023-03-27

**Authors:** Manni Mo, Bo Fu, Piyush Hota, Pinar Cay-Durgun, Ran Wang, Edward H. Cheng, Peter Wiktor, Francis Tsow, Leslie Thomas, Mary Laura Lind, Erica Forzani

**Affiliations:** 1 Health Futures Center, Arizona State University, Phoenix, AZ 85054, USA; 2 Center for Bioelectronics and Biosensors, Biodesign Institute, Arizona State University, Tempe, AZ 85287, USA; 3 Division of Nephrology, Mayo Clinic, Scottsdale, AZ 85259, USA; 4 School for Engineering of Matter, Transport and Energy, Arizona State University, Tempe, AZ 85287, USA

**Keywords:** colorimetric sensing, tunable response, continuous sensing, sensor saturation, internal diffusion, automated sampling, artificial intelligence

## Abstract

Colorimetric sensors are widely used because of their inherent advantages including accuracy, rapid response, ease-of-use, and low costs; however, they usually lack reusability, which precludes the continuous use of a single sensor. We have developed a threshold-responsive colorimetric system that enables repeated analyte measurements by a single colorimetric sensor. The threshold responsive algorithm automatically adjusts the sensor exposure time to the analyte and measurement frequency according to the sensor response. The system registers the colorimetric sensor signal change rate, prevents the colorimetric sensor from reaching saturation, and allows the sensor to fully regenerate before the next measurement is started. The system also addresses issues common to colorimetric sensors, including the response time and range of detection. We demonstrate the benefits and feasibility of this novel system, using colorimetric sensors for ammonia and carbon dioxide gases for continuous monitoring of up to (at least) 60 detection cycles without signs of analytical performance degradation of the sensors.

## 1. Introduction

Colorimetric sensing can quantify target analytes by detecting color changes associated with chemical reactions between the analytes and the corresponding sensor materials [1,2,3], enabling in some cases a remarkable selectivity [4,5,6], low cost, and rapid response times [1,2,3]. A plethora of colorimetric sensors have been applied to detect environmental pollutants [7,8], toxic agents [9,10,11], biomarkers, and pharmaceutical- and food-relevant molecules [12,13,14]. Along this line, several reviews have been published [15,16,17,18,19]. Various detection systems can provide the readout of colorimetric sensors, including simple visual color interpretations, hand-held reading devices [20,21], commercial spectrometers [22,23], custom embedded systems [24,25], and complementary metal-oxide semiconductor (CMOS) chips embedded in commercial cameras and smartphones [15,26,27,28,29,30].

Despite these reports, further research is required to develop colorimetric sensors that maintain high selectivity while operating continuously with repeated measurements performed over time. Reversible colorimetric sensors (e.g., colorimetric electric noses) have achieved the detection of volatile organic compounds via pattern recognition [31,32]. The colorimetric electronic noses use non-selective adsorption reactions on organic dyes and require an array of multiple colorimetric sensors.

Here, we introduce for the first time a new system that achieves the continuous and automated monitoring of a specific analyte using a single colorimetric sensor with a seamless integration of optoelectronics and a threshold-responsive algorithm.

Different strategies to prolong the lifetime of colorimetric sensors have been used. Zhou et al. made a membrane-based colorimetric chlorine sensor for continuous chlorine gas monitoring for an extended period with a specially designed membrane to control the diffusion of the gas, which prevented rapid sensor saturation [33]. Sotirov et al. built a metal-organic framework based on a trimesic acid and Co^2+^ colorimetric sensing material for ammonia (NH_3_) detection; they showed sensor reusability by applying an acidic process to regenerate the sensor [34]. Wang et al. reported a microfluidic-colorimeter nitric oxide (NO_2_) gas sensor for continuous NO_2_ monitoring for longer periods (i.e., for hours when exposed to parts per billion concentrations). This system used the full length of a microfluidic-channel (mm length) and a complementary metal-oxide-semiconductor (CMOS) imager as the detector to extract absorbance changes over time along the channel length for continuous monitoring [2]. Lin et al. applied the same methodology to the simultaneous detection of ozone, NO_2_, and formaldehyde gases [1,35]. Lin et al. also showed continuous monitoring of carbon monoxide (CO) in a single sensor by limiting the exposure of the sensing probe to the gas [9]. Despite the promising results from this prior work, the challenge of a limited sensor lifetime in single sensor systems persists.

We introduce a new system, named the “Threshold Responsive Colorimetric Sensing System”. The system is empowered by a responsive algorithm that utilizes sensor signal changes to protect the saturation of a colorimetric sensor; this was inspired by a method initially introduced by the members of our team, Tsow, Forzani, and Tao [36], with quartz crystal tuning fork sensors and reactions based on weak analyte-binding site interactions. Here, we present the concept of specific bindings in colorimetric reactions. The threshold-responsive algorithm adjusts the exposure of the sensor to an analyte and prevents the sensor binding sites from becoming fully saturated by switching the feed to purging air once the sensor reaches the threshold value. In addition, the responsive algorithm is based on the rate of the sensor signal change rather than the sensor signal change, which results in response times of seconds rather than minutes and avoids sensor response drift. In this study, we evaluated the system performance based on the hysteresis, sensitivity, and the number of sensor reusability cycles [37].

## 2. Materials and Methods

### 2.1. Principle of the Threshold-Responsive Colorimetric Sensing System

**Figure 1:**
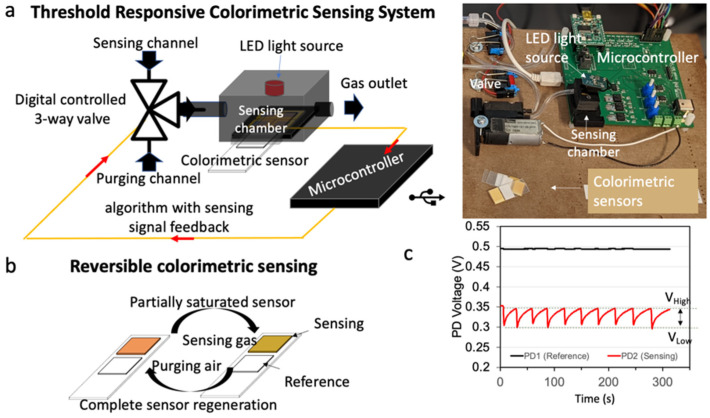
Schematic of the threshold-responsive colorimetric sensing system. (**a**) It has a chemical and mass transport system, optoelectronics system, and a threshold-responsive algorithm embedded in the microcontroller. The chemical and mass transport system includes a 3-way solenoid gas valve, an air pump for analyte and analyte-free air injection, and a sensing chamber. The sensing chamber contains a light emitting diode (LED) and a colorimetric sensor. The optoelectronics system has two photodiodes which capture signals from the reference (PD1: reference) and the sensing (PD2: sensing) areas of the sensor. The microcontroller algorithm controls the valve and pump, and it transfers data to a portable computer (PC). Insert: picture of the system. (**b**) Reversible colorimetric sensing is achieved by controlled analyte exposure to the sensor and complete sensing surface regeneration (**c**). An example of the threshold-responsive algorithm as described in the section: principle of the threshold-responsive colorimetric sensing system.

Figure 1a shows a schematic representation of the new threshold-responsive colorimetric sensing system designed for gas detection, which enables repeated colorimetric sensing using a single sensor by controlling the analyte exposure and completing the sensing surface regeneration. The system uses two channels connected to a colorimetric sensor inserted in a sensing chamber: a “sensing channel” that provides the gas analyte sample inlet, and a “purging channel” that provides a clean air inlet. Sensor exposure to a gas analyte or clean air is regulated using a 3-way gas valve. A microcontroller controls the valve and affects the alternation of the connection of either channel to the sensing chamber; the precise duration and frequency of the alteration between channels are based on the sensor signal and the threshold-responsive algorithm stored in the microcontroller firmware.

In this described application, the sensor (Figure 1b) has a sensing area, which is modified with a gas sensing probe on a solid substrate, and a reference area, formed by the unmodified solid substrate. The sensor response is detected using a light emitting diode (LED) located on the superior aspect of the sensing chamber (e.g., above the sensor) and two photodiodes. The photodiodes are located on the inferior aspect of the sensing chamber (e.g., below the sensor’s sensing area and the sensor’s reference area, respectively). When a sensor reaction occurs and creates a color change, the voltage of the sensing area (Vs) changes while the voltage of the reference (Vr) area remains unchanged (Figure 1c). Our threshold-responsive algorithm in the microcontroller firmware calculates the “output voltage ratio” as Vs/Vr. The algorithm uses the absolute Vs/Vr change (|Vs/Vr| change) as feedback to adjust the time the analyte gas is exposed to the sensor. The |Vs/Vr| change is preset, and the preset value implies that the Vs (the only voltage changing during sensing) will be allowed to reach: 1—a “threshold low” (V_low_), which corresponds to the baseline Vs (in the absence of analyte), and 2—a “threshold high” (V_high_), which corresponds to the maximum allowable limit of the sensor voltage response. Because the “output |Vs/Vr| change” is an absolute value, the V_high_ can be: 1—higher than the V_low_ when the sensor becomes lighter in response to the analyte (e.g., purple/blue to green/yellow) or 2—lower than the V_low_ when the sensor becomes a darker color in response to the analyte (e.g., orange/yellow to dark green). The firmware includes an “if” and “then” function, where the analyte gas exposure of the sensor occurs for any condition where the Vs and Vr values have a |Vs/Vr| change ≤ preset threshold. When the condition is no longer met, the gas exposure of the sensor is stopped by switching the gas valve to the exposure for clear air (i.e., the purging channel).

In a typical sensing cycle, first, the 3-way valve is open which pumps the gas analyte through the sensing channel and into the sensing chamber; this causes the sensor to change color in response to the concentration of analyte present in the gas. Simultaneously, the microcontroller continuously calculates the |Vs/Vr|, the instantaneous output voltage ratio. When the output voltage ratio reaches the preset |Vs/Vr| change value (V_high_), the microcontroller actuates the 3-way valve, which switches from the sensing channel to the purging channel and brings clean air (i.e., analyte-free air); this promotes the regeneration of the sensor surface’s baseline status. The microcontroller actuates the 3-way valve again when the output voltage ratio reaches the preset V_low_ and starts another measurement cycle (i.e., a repeated gas analyte injection). Therefore, data collected using the responsive algorithm, with a preset |Vs/Vr| change (V_high_ and V_low_), leads to an oscillation of the sensor voltage response as the system switches from delivering analytes or purging air. Voltages are used to process the sensor signal as absorbance: absorbance = −log (Vs/Vr). The slope defined by the absorbance change over time (∂absorbance/∂t = ∂ (−log (Vs/Vr))/∂t) during the analyte injection is then correlated to the concentration of the gas analyte (i.e., calibration). Therefore, the absorbance change rate is the sensor system’s post-processed signal.

### 2.2. Details of the Optoelectronics System

The threshold-responsive colorimetric sensing system contains five major parts including: 1—the 3-way solenoid gas valve (Parker); 2—a direct current (DC) motor air pump (Parker, T3EP-1ST-05-3FFP) for the analyte and clean air injection; 3—a sensing chamber with LED; 4—a reversible colorimetric sensor (located within the sensing chamber); and 5—a printed circuit board (PCB) with two photodiodes and a microcontroller (PIC32MX450F512H, microchip) that controls the 3-way valve, controls the pump, and transfers data to a portable computer (PC). All electronic components including the driver for the 3-way valve, the driver for the air pump, the microcontroller, and the data transferring circuit are soldered onto the custom PCB. The sensing chamber (Figure 1) receives the colorimetric sensor, and it is connected to the 3-way valve, which alternates an injection of gas analyte and analyte-free air (from a sampling channel connected to a gas analyte sample and a purging channel connected to an air source, respectively). For the tests reported in this paper, we delivered the gas samples from Mylar^®^ bags (Custom Sensor Solutions, Oro Valley, AL, USA) prepared with precise dilutions of the gases from the master supply, and the purging air from the atmosphere was delivered into the chamber through the inlet using an air pump via polytetrafluoroethylene tubing (Tygon^®^ SE-500) (McMaster-Carr, Elmhurst, IL, USA) and a zeroing filter containing activated carbon (Purafil, Atlanta, GA, USA).

The sensor’s color changes were captured by the two photodiodes on the custom PCB with one photodiode dedicated to collecting the transmitted light from the sensor’s sensing area and one photodiode dedicated to collecting transmitted light from the sensor’s reference area. Figure 1c shows an example of the output voltage of the sensing and reference photodiodes. This data is transferred from the microcontroller to a PC using a USB cable and the UART protocol with a time-resolution of 250 ms. In addition, we built a custom MATLAB App Designer program to receive and save the collected photodiode voltage data on the PC. Except for the PC, the entire colorimetric sensing system was powered by a 12 V power supply.

### 2.3. Colorimetric Sensors

Previously, Yan et al. developed a colorimetric filter paper for reversible pH detection in liquids using chemiluminescent luminol derivatives with ultraviolet (UV)-visible absorption spectra [38]. To demonstrate the concept of a reversible colorimetric sensor response, we hypothesized that protonation and deprotonation reactions could be applied to detect basic and acidic small molecule gases. Therefore, we applied the threshold-responsive colorimetric sensing system concept to detect ammonia (NH_3_) and carbon dioxide (CO_2_).

As described in our previous publications, we prepared colorimetric sensors for NH_3_ [39] and CO_2_ [40]. The NH_3_ sensors were prepared from Bromophenol Blue (BpB). BpB appears yellow/orange at pH < 3 and appears blue at pH > 4.6; from pH 3–4.6, BpB transitions through shades of yellow/orange and green/blue. Because NH_3_ is an alkaline gas, our BpB-based sensors turned blue as NH_3_ passed through the sensing chamber and over the sensor surface [41]. We prepared the CO_2_ sensors using m-cresol purple. M-cresol purple appears purple/blue at pH > 9 and green/yellow at pH < 7.4. Because CO_2_ is an acidic gas, our m-cresol purple-based sensors turned green/yellow as CO_2_ passed through the sensing chamber and over the sensor surface.

### 2.4. Gas Sample Preparation

All concentrations were represented in volume per volume concentrations either in part-per-million (ppm) or percentage (%). We purchased standard calibration gases from Calibration Technologies, Inc. (Columbia, MO, USA) of 20,000 ppm (2%) NH_3_ gas and a 4% standard CO_2_ gas. We prepared NH_3_ gas concentrations from 10 ppm to 1000 ppm and CO_2_ gas concentrations from 0.5% to 4% by diluting the standards with filtered ambient air. The calibration gas and clean air were directed into a 40 L Tedlar^TM^ (Custom Sensor Solutions) bag using a manual switching valve and a 12 V diaphragm gas pump for a predetermined time ratio to reach the desired final gas concentration.

### 2.5. System Tests and Environmental Testing Conditions

We performed tests in which we delivered different gas concentrations to the system and observed the system response using our threshold-responsive algorithm (with the switching determined by the |Vs/Vr| change (e.g., V_high_ and V_low_)) and a conventional time-based algorithm (in which the sampling and purging periods are based on fixed time lengths).

All the experiments were performed at temperatures and humidity levels between (22–23) °C and (30–40)%, respectively. For the hysteresis experiments, all the experiments were performed within an hour in a temperature- and humidity-controlled lab.

## 3. Results

### 3.1. Characterization of the Threshold-Responsive Colorimetric Sensing System

To demonstrate the capacity of our new system for the continuous monitoring of analytes, we tested the hysteresis of the sensor response. Hysteresis is defined as the difference between separate measurements of the same analyte concentration occurring within a series of increasing and decreasing analyte concentration exposures [42].

In principle, the system activates the sensor purging mode to protect the sensor. With this mechanism, we expect the sensor to be protected from complete saturation and, thus, be able to be thoroughly and quickly regenerated during the purging period. Therefore, different analyte concentrations should provide consistent sensor responses regardless of the sequence in which the various analyte concentrations are introduced to the sensor (hysteresis).

**Figure 2:**
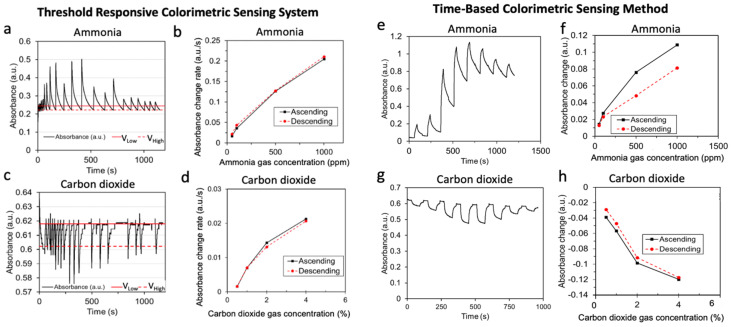
Hysteresis measurement of the responsive colorimetric sensing system (**a**–**d**) and time-based sensing method (**e**–**h**) conducted with 50 to 1000 ppm ammonia (**a**,**b**,**e**,**f**) and 0.5 to 4% carbon dioxide (**c**,**d**,**g**,**h**). (**a**,**c**) represent the raw sensor response with a preset output voltage ratio threshold (Vs/Vr) change of 0.09 associated with a low (V_Low_) and a high (V_High_) sensing voltage threshold.

Figure 2a,c shows the response of the threshold-responsive sensing system over time for NH_3_ and CO_2_ sensors, respectively. In these tests, the system was preset to trigger the purging mode if the change in the voltage ratio (Vs/Vr) was 0.09. In addition, the sensors were exposed to increasing analyte concentrations followed by decreasing analyte concentrations to evaluate the sensor response hysteresis feature. Figure 2b,d shows the corresponding calibration curves resulting from the system’s response analysis for the NH_3_ and CO_2_ sensors, respectively. The same 610 nm LED light source was used in the sensor chamber for both the NH_3_ and CO_2_ colorimetric sensors. At this wavelength, the absorbance increased as the NH_3_ sensor changed color from yellow/orange to green during NH_3_ exposure. In contrast, the absorbance decreased as the CO_2_ sensor color changed from blue/purple to green during CO_2_ exposure. Additionally, we observed an overshoot effect in the sensor signal greater than the preset V_high_ threshold; we hypothesize this measured overshoot is the result of analyte accumulation inside the sensing system (e.g., the tubing and pump) at the time of the valve switching and subsequent transport to the sensing chamber that is pushed by the purging air.

Regardless of the overshoot effect, the absorbance change rate (a.u./s) measured for each analyte concentration showed a reproducible response independent of the history of the sensor exposure; e.g., no hysteresis. This is an important feature of the new responsive colorimetric sensing system, which enables reproducible continuous monitoring.

For comparative purposes, we studied the system response for the same NH_3_ and CO_2_ sensors using the conventional time-based algorithm. In this method, the system cycled the sensor exposure to the analyte gas for a fixed amount of time (60 s) and purged air for a fixed amount of time (30 s). Figure 2e–h shows the results. Figure 2e,g shows that the sensor baseline response changed over time due to an incomplete recovery of the initial number of unoccupied binding sites in the fresh sensor. In addition, Figure 2f shows the significant hysteresis effect in the calibration curve for NH_3_ (e.g., ~40% change for 500 ppm concentration), which is unacceptable for continuous monitoring applications. Figure 2h shows the calibration curve for the CO_2_ sensor, which has less hysteresis (e.g., 10% for 1% CO_2_) than the NH_3_ sensor (Figure 2f) due to the intrinsic nature of the fast desorption of this specific colorimetric sensing probe [40]; however, the hysteresis shown for the conventional time-based method for the CO_2_ sensor (Figure 2h) is larger than the corresponding one for our responsive algorithm (Figure 2d). Overall, the threshold-responsive-based approach resulted in more optimal hysteresis characteristics for the implementation of continuous analyte monitoring compared to the time-based sensing method.

### 3.2. Reproducibility Performance of the Threshold-Responsive Colorimetric Sensing System for Continuous Monitoring

**Figure 3:**
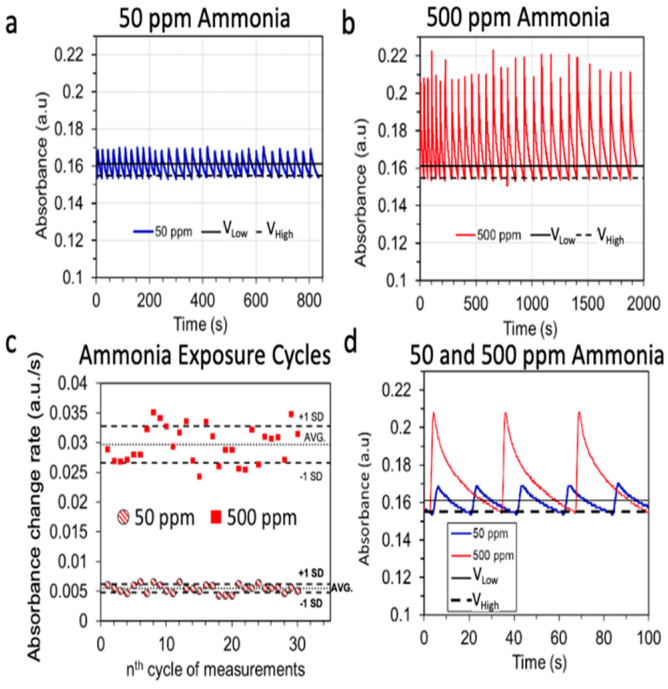
Continuous monitoring capacity of the threshold-responsive colorimetric sensing system. (**a**,**b**) Absorbance of a single ammonia sensor over time upon consecutive cycling exposure of 50 and 500 ppm NH_3_ samples. (**c**) Corresponding ammonia sensor response (absorbance change rate) to 50 and 500 ppm NH_3_ of 30 continuous sensing-purging cycles of the single ammonia sensor from (**a**,**b**). (**d**) Snapshot of 100 s of sensing for continuous detection of 50 and 500 ppm NH_3_ (**a**,**b**). (**a**,**b**,**d**) show the low (V_Low_) and high (V_High_) sensing voltage threshold associated with a preset voltage ratio (Vs/Vr) change of 0.09.

To further demonstrate the potential of the threshold-responsive algorithm for the continuous monitoring of analytes, we exposed the NH_3_ sensor (which had the most hysteresis in the conventional time-based method) to low (50 ppm) and high concentrations (500 ppm) of NH_3_ for 60 consecutive measurement cycles. Furthermore, we tested the reproducibility of the sensor response. Figure 3 shows an example of the threshold-responsive-based sensor response to 30 consecutive sensing cycles of 50 ppm (Figure 3a) and 30 consecutive sensing cycles of 500 ppm (Figure 3b) of NH_3_ gas assessed using the same sensor when the low and high concentrations were alternated every 10 detection cycles (Figure 3d). Figure 3a–c shows a reliable restoration of the baseline for each sensing cycle. In addition, the variations in the absorbance change rate calculated for each measurement fell mostly within ±1 standard deviation of the average value (Figure 3c), which was (0.030 ± 0.003) a.u./s for 500 ppm NH_3_ and (0.0050 ± 0.006) a.u./s for 50 ppm NH_3_. Therefore, we can reasonably conclude that the threshold-responsive-based algorithm prevented the saturation of the NH_3_ sensor, thus enabling continuous monitoring and reusability of the sensor.

### 3.3. Comparative Analysis of the Sensing Parameters of the Threshold-Responsive Colorimetric Sensing System

**Figure 4:**
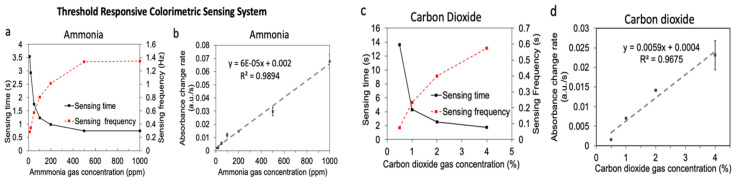
Comparative analysis of the sensing parameters of the threshold-responsive colorimetric sensing system: sensing time, sensing frequency, and absorbance change rate of ammonia gas (**a**,**b**) and carbon dioxide gas (**c**,**d**) at different concentrations. Error bars show the standard deviation of triplicate measurements on a single sensor. Note: for some measurements the bars are smaller than the symbol and not visible.

The threshold-responsive colorimetric sensing system provides a response that could be calculated in the frequency domain instead of the amplitude domain. For instance, within each sensing cycle, there is a “sensing time” that is inversely proportional to the concentration of the analyte—the lower the analyte concentration, the higher the “sensing time” since it takes longer for the output |Vs/Vr| change to reach the preset value. The left axis of Figure 4a shows that the NH_3_ sensing time ranges from 3.5 s to 0.5 s for samples with NH_3_ concentrations ranging from 10 ppm to 1000 ppm. The left axis of Figure 4c shows that the CO_2_ sensing time ranges from 13 s to 2 s for samples with CO_2_ concentrations ranging from 0.5% to 4%.

In addition, the reciprocal of “sensing time” is “sensing frequency”, which can be accounted for as a sensing parameter for the threshold-responsive system’s algorithm as well (Figure 4a,c, right axis). There is a proportional relationship between “sensing frequency” and analyte concentration. Moreover, Figure 4b,d shows the maximum absorbance rate change for the same ammonia and carbon dioxide sensors, whose “sensing time” and “sensing frequency” responses are shown in Figure 4a and Figure 4c, respectively. Meanwhile, the sensing times and sensing frequencies may be easier to acquire than the calculation process required by a maximum absorbance rate change, accounting for the absorbance change rate being convenient to calibrate with a linear proportionality up to 1000 ppm NH_3_ and 4% CO_2_, respectively; thus, avoiding a saturation of the sensor signals.

Notably, repetitions of the gas sample analysis rendered coefficients of variation smaller than 10% for most of the concentrations shown in Figure 4b,d. Greater standard deviations were found for the slopes of relatively-high concentration samples, including 500 ppm and 1000 ppm NH_3_ and 4% CO_2_. This variability was due to the measurement resolution. A way to overcome the problem could be by presetting a |Vs/Vr| change to a larger value. Presetting different voltage ratio changes is feasible and could also be automatically incorporated into the system microcontroller’s threshold-responsive algorithm.

### 3.4. Performance of the Threshold-Responsive Colorimetric Sensing System Using Different Preset Threshold Conditions

**Figure 5:**
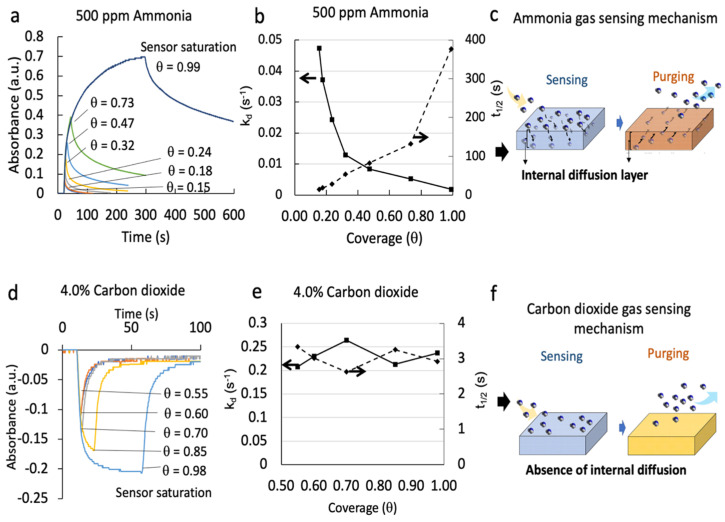
Kinetics study of the threshold-responsive colorimetric sensing system at different conditions of the sensor coverage (θ) determined by the preset value of the |Vs/Vr| change of the system. (**a**,**d**) Absorbance changes over time for association and dissociation of ammonia (**a**) and carbon dioxide (**d**). (**b**,**e**) Corresponding calculated dissociation kinetics constants (k_d_) and half-life time (t_1/2_) for ammonia (**b**) and carbon dioxide (**e**). (**c**) A diagram showing how internal diffusion of the gas analyte to the inner layer of the sensor (**c**) can lead to prolonged purging time and decreased dissociation kinetic constants. (**f**) A diagram showing how the lack of internal diffusion of gas analyte (which in the carbon dioxide sensor is achieved by a phase transfer catalyst) allows complete purging for each detection cycle, which leads to fast dissociation constants regardless of the degree of the sensor coverage. (**b**,**e**) Arrows indicate the corresponding y-axis for k_d_ and t_1/2_ values. (**a**,**d**) Lines are used to guide the eye and connect a θ value with the corresponding curve.

To characterize the analyte measurement resolution, e.g., how frequently the analytes could be measured by the threshold-responsive colorimetric sensing system, we analyzed the effect of increasing the preset |Vs/Vr| change on the desorption kinetic constants of the NH_3_ and CO_2_ sensors. This translates for: 1—an ammonia sensor decreasing the threshold high value, V_high_ (e.g., increasing incursions to darker green colors from the yellow/orange initial color), and 2—a carbon dioxide sensor increasing the threshold high value, V_high_ (e.g., incursions to lighter green/yellow colors from the purple/blue initial color), which means more of the sensor’s binding sites are occupied before the sensor is flushed with purged air and recovered to the baseline. This increase in sensor coverage (θ) increases the potential degradation of the sensor capacity for reversible continuous monitoring of the analyte. Figure 5a,c shows the absorbance vs. time profiles from our threshold-responsive colorimetric sensing system with |Vs/Vr| changes under constant ammonia and carbon dioxide concentrations, respectively. Assuming a first order desorption process [43]:

*k_d_*
(analyte-sensing probe complex) → analyte + sensing probe(1)

With *k_d_* representing the dissociation kinetic constant of the sensing reaction product (analyte-sensing probe complex), and *t*_1/2_ representing the reaction product half-life time (−ln 0.5/*k_d_*) [44], where the *k_d_* and corresponding *t*_1/2_ were calculated.

Figure 5b,d shows the resulting dissociation kinetic constants for NH_3_ and CO_2_, respectively, as a function of θ at a fixed analyte concentration. The NH_3_ sensor exhibits a significant dependence on the dissociation kinetics with θ. The dissociation kinetic constant decreased significantly for θ between 0.20 and 0.99. On the contrary, the CO_2_ sensor presented values of dissociation kinetic constants and half-life times that were independent of the sensor coverage. This is because a phase transfer catalyst (a quaternary ammonium hydroxide) was added to the CO_2_ sensor, enabling a rapid proton-exchange and accelerating the desorption process (Figure 5f). In contrast, the NH_3_ sensor desorption process was increasingly slower with a higher θ. We hypothesize that the lack of a phase transfer catalyst in the ammonia sensor created an additional resistance to analyte desorption from the BpB sensing probe, which involved the internal diffusion of NH_3_ within the sensing layer [43], as illustrated in Figure 5c. From an application perspective, we desire the purging time to be as short as possible to enable essentially continuous measurements (e.g., multiple consecutive measurements at short intervals). For this reason, the |Vs/Vr| ratio change should be selected to balance the desired sensitivity, dynamic range, and resolution of the measurements. Currently, a |Vs/Vr| change of 0.7 to 0.9 is suitable for detecting NH_3_ from 50 ppm to 1000 ppm with a measurement resolution of < 6 min. More specifically, the threshold-responsive colorimetric sensing system has a final throughput of at least 33 measurements in 14 min for low ammonia and CO_2_ concentrations (50 ppm and 1%, respectively) and of ~28 measurements in 29 min for high ammonia and CO_2_ concentrations (500 ppm and 4%, respectively).

### 3.5. Analysis of the Overshoot Response of the Threshold-Responsive Colorimetric Sensing System

**Figure 6:**
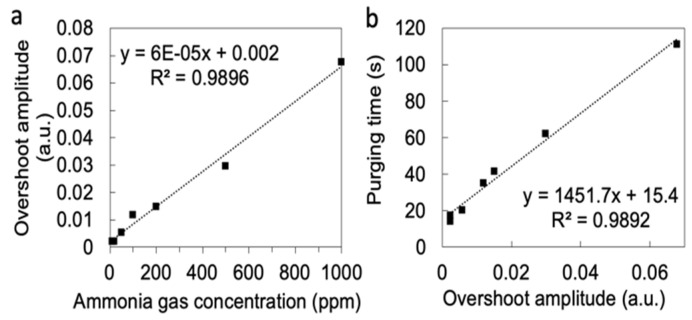
(**a**) Overshoot amplitude of ammonia gas vs. ammonia gas concentration. (**b**) Purging time vs. overshoot amplitude.

For each measurement cycle using the threshold-responsive sensing system, the output response exceeded the values corresponding to the preset |Vs/Vr| change threshold. This phenomenon is the so-called “overshoot effect” and can be quantified by evaluating the sensor signal amplitude (e.g., the signal change from the baseline to the maximum). Figure 6a shows the overshoot absorbance amplitude as a function of the ammonia gas concentrations. As can be observed in Figure 6a, the overshooting absorbance amplitude is directly proportional to the NH_3_ concentration, and we hypothesize that, to some degree, this results from analyte storage in the dead space volumes within the system and absorption to the interior surfaces of the system (e.g., within the tubing and pump). In addition, Figure 6b shows the directly-proportional relationship between the overshoot amplitude and analyte purging time, indicating that the overshooting phenomenon increases the time required for the sensor to return to its baseline state during purging due to the increased sensor saturation.

Overshooting is undesirable in colorimetric sensors because the increased number of occupied sensor binding sites will increase the purging time of the sensor, making it less reliable to make measurements of the concentration by simply counting the cycles over a period directly. Our threshold-responsive system automatically adjusts the duration of the sensing time and purging time to allow the baseline sensor conditions to be reached before initiating the next measurement cycle. As demonstrated here, reliable continuous analyte monitoring for up to (at least) 60 cycles is possible regardless of the overshooting effect. This demonstrated that a threshold-based algorithm combined with an optoelectronic sensing method shows that colorimetric sensors have the potential for continuous use. Colorimetric sensors may offer a more cost-effective solution than other methods requiring more dedicated, specialized facilities (e.g., microfabrication/cleanrooms) for mass production.

## 4. Conclusions

This study investigated the effect of sensor saturation on the dissociation kinetic constants and capacity of sensor regeneration. We developed a threshold-responsive algorithm that limits the maximum sensor absorbance change and prevents excessive saturation of the sensor binding sites. Compared to conventional time-based sensing algorithms, the incorporation of a threshold-responsive algorithm to reflexively control the analyte-sensor exposure time based on real-time sensor status can eliminate hysteresis effects and achieve more reproducible analyte measurements for at least 60 cycles using the same NH_3_ and CO_2_ colorimetric sensors. These data show for the first time the reliable reusability of colorimetric sensors working with specific reactions for continuous measurement.

Our threshold-responsive colorimetric sensing system provides a solution for continuous and reproducible measurements of a gas analyte on a colorimetric sensor. Given the plethora of single-use colorimetric sensors [15,26,27,28,29,30] that demonstrate the specific detection of environmental pollutants [7,8], toxic agents [9,10,11], biomarkers, and pharmaceutical- and food-relevant molecules [12,13,14], the implementation of the presented sensing system enables the continuous monitoring of analytes that could be integrated into wireless communication networks [17]. In addition, the threshold-responsive-based sensing approach may also be applicable to other sensing systems, such as surface plasmon resonance sensors [45], electrochemical sensors [46], fluorescent sensors, and other acoustic sensors [47], different from tuning fork sensors where the concept was demonstrated originally for weak adsorptive interactions. Furthermore, the relevance of the threshold-responsive colorimetric sensing system may be even more significant for applications where the sensor costs are crucial [37].

Finally, the new sensing system’s ability to offer a versatile way to preset the degree of sensor saturation via output voltage ratio threshold limits offers a convenient tool to study the desorption kinetics of a gas molecule and a solid specific binding site in colorimetric reactions.

## Data Availability

For data availability, contact the corresponding author: Erica Forzani.

## Abbreviations

PD: photodiode; LED: light emitting diode; PCB: printed circuit board; PC: portable computer; BPB: bromophenol blue.
